# Major component analysis of dynamic networks of physiologic organ interactions

**DOI:** 10.1088/1742-6596/640/1/012013

**Published:** 2015-09-28

**Authors:** Kang K. L. Liu, Ronny P. Bartsch, Qianli D. Y. Ma, Plamen Ch. Ivanov

**Affiliations:** 1 Department of Physics, Boston University, 590 Commonwealth Ave, Boston, MA 02215, USA; 2 Department of Neurology, Beth Israel Deaconess Medical Center, Harvard Medical School, Boston, MA 02115, USA; 3 Department of Physics, Bar-Ilan University, Ramat-Gan, 52900, Israel; 4 College of Geographical and Biological Information, Nanjing University of Posts and Telecommunications, Nanjing, China; 5 Division of Sleep Medicine, Brigham and Women’s Hospital, Harvard Medical School, Boston, MA 02115, USA

## Abstract

The human organism is a complex network of interconnected organ systems, where the behavior of one system affects the dynamics of other systems. Identifying and quantifying dynamical networks of diverse physiologic systems under varied conditions is a challenge due to the complexity in the output dynamics of the individual systems and the transient and non-linear characteristics of their coupling. We introduce a novel computational method based on the concept of time delay stability and major component analysis to investigate how organ systems interact as a network to coordinate their functions. We analyze a large database of continuously recorded multi-channel physiologic signals from healthy young subjects during night-time sleep. We identify a network of dynamic interactions between key physiologic systems in the human organism. Further, we find that each physiologic state is characterized by a distinct network structure with different relative contribution from individual organ systems to the global network dynamics. Specifically, we observe a gradual decrease in the strength of coupling of heart and respiration to the rest of the network with transition from wake to deep sleep, and in contrast, an increased relative contribution to network dynamics from chin and leg muscle tone and eye movement, demonstrating a robust association between network topology and physiologic function.

## 1. Introduction

A defining feature of the human organism is the presence of complex signaling and interaction processes between organ systems and sub-systems. These interactions occur at multiple levels of integration and across spatio-temporal scales to optimize and coordinate physiologic organ functions. Coordinated network interactions of organ systems are essential to maintain health and generate distinct physiologic states, e.g., rest and exercise; wake and sleep; light and deep sleep; dreams; consciousness and unconsciousness. Disrupting these communications can lead to dysfunction of individual systems or to a collapse of the entire organism.

A fundamental question is how organ systems dynamically interact and collectively behave as a network to produce health or disease. A system-wide integrative approach is needed to address this fundamental question. We have recently initiated a new area of research, Network Physiology[1, 2], with the aim to uncover the nature and basic characteristics of organ-to-organ interactions, and to associate specific networks of organ interactions with physiologic function during distinct states and conditions.

Identifying and quantifying organ interactions is a major challenge due to several levels of complexity inherent to the dynamics of organ systems: (i) each organ is a multi-component system with its own regulatory mechanisms, leading to complex emerging behavior characterized by intermittent, scale-invariant and nonlinear output signals [3, 4]; (ii) organ systems have different types of output dynamics — oscillatory, stochastic or mixed [5, 6]; (iii) systems operate on different time scales from msec to hours; (iv) the output dynamics and coupling links between systems vary in time and with different physiologic states [7, 8, 9, 10]; (v) as we have recently discovered certain pairs of organ systems can communicate through several forms of coupling that switch on/off and can simultaneously coexist [11, 12, 13]; (vi) due to non-linear feedback interactions, global network dynamics of the entire organism can not be simply expressed as a sum of the behaviors of individual systems, and can be strongly influenced by minor changes in the relative strength of systems’ interactions, even when the network topology between these systems remains unchanged.

Currently, there are no analytic instrumentarium and theoretical framework suitable to probe diverse organ systems with different output dynamics, and to quantify their interactions from continuous streams of noisy and transient signals. The current framework of complex network theory is also not directly applicable to address challenges in this new field, since it mainly addresses (i) network growth or disintegration by adding/removing nodes and links, or (ii) the flow of information on networks with fixed structure. The fundamental question we address in the field of Network Physiology relates to a different aspect, namely, to characterize how global network behavior and function emerge from complex output dynamics and coupling interactions between diverse systems (nodes), and how global behavior changes in response to variations in the strength of interactions (links), even when the topology of the network remains unchanged.

We propose a novel approach based on the concept of time delay stability, to identify and quantify pair-wise interactions between diverse systems (network nodes) with different types of output dynamics, combined with a major component analysis designed to dissect complex networked interactions between multiple organ systems.

## 2. Methods

### Time Delay Stability (TDS):

The structural and neuronal networks that control physiological systems lead to a high degree of complexity, which is further compounded by various coupling and feedback interactions that continuously vary in time, and the nature of which is not understood. These systems operate on different time scales, from msec to hours, and exhibit multiple coexisting forms of coupling. To quantify these interactions and characterize how they change in time under different physiological conditions, we study the time delay with which modulations in the output dynamics of a given system are consistently followed by corresponding modulations in the signal output of another system (time delay stability, TDS). Periods with constant time delay indicate a stable physiological interaction, and stronger coupling between systems results in longer periods of TDS.

Physiological output signals are first re-sampled at 1 Hz and normalized to zero mean and unit standard deviation within overlapping time windows of ∆*t* = 60s with a moving step of 30 seconds (effectively de-trending the signals). Synchronous bursts in the normalized signals lead to pronounced cross-correlation calculated in windows of ∆*t* = 60s in steps of 30*s*. The time delay *τ*_0_ is determined by the position of the maximum of the cross-correlation function in each moving window ∆*t*. We identify two systems as linked if their corresponding signals exhibit a time delay that does not change by more than *±*1 sec for several consecutive time windows ∆*t*. We track the values of *τ*_0_ along the series {*τ*_0_}∆*t* — when for at least four out of five consecutive time windows ∆*t* (corresponding to a period of 5 × 30 sec) the time delay remains in the interval [*τ*_0_ *−* 1,*τ*_0_ + 1] these segments are labeled as stable. The procedure is repeated for a sliding time window with a step size one along the entire series {*τ*_0_}∆*t*. Longer periods of TDS between the output signals of two systems reflect more stable interaction/coupling between these systems. Thus, the strength of coupling is determined by the percentage of time when TDS is observed: higher percentage of TDS corresponds to stronger coupling. The % TDS is calculated as the fraction of segments with stable time delay out of the entire time series {*τ*_0_}∆*t*.

### Major Component Analysis (MCA):

To dissect the complex network of physiologic organ interactions and to identify (i) which subset of links play major role and (ii) what is the relative contribution of individual organs (nodes) to the entire network dynamics, we employ matrix theory and the concept of Major Component Analysis. Utilizing the matrix of network interactions, where each matrix element represents the pair-wise coupling between organ systems as measured by the % TDS, we perform matrix diagonalization to extract the spectrum of eigenvalues and associated eigenvectors, where each eigenvector element represents the relative contribution of each organ to the corresponding eigenmode of the network. The global network dynamics is thus a linear superposition of the different eigenmodes.

First, we obtain the TDS matrix of pair-wise interactions, where matrix element *A*_*i,j*_ represents the strength of dynamic coupling between organ *i* and organ *j*, measured by the % TDS derived from the output signals of these two systems.

Matrix *A* is symmetric and can be diagonalized to determine its spectrum of real eigenvalues.

(1) $$D\equiv U\;AU^{T}=\left(\begin{array}{c} V^{1}\\ V^{2}\\ \vdots \\ V^{n} \end{array}\right)\left(\begin{array}{cccc} A_{1,1} & A_{1,2} & \cdots & A_{1,n}\\ A_{2,1} & A_{2,2} & \cdots & A_{2,n}\\ \vdots & \vdots & \ddots & \vdots \\ A_{n,1} & A_{n,2} & \cdots & A_{n,n} \end{array}\right)\left(\begin{array}{cccc} V^{1T} & V^{\text{2}T} & \cdots & V^{nT} \end{array}\right)=(\begin{array}{cccc} {\text{λ}}_{1} & 0 & \cdots & 0\\ 0 & {\text{λ}}_{2} & \cdots & 0\\ \vdots & \vdots & \ddots & \vdots \\ 0 & 0 & \cdots & {\text{λ}}_{{}^{n}} \end{array}),$$

where U is a unitary matrix; λ*_i_* is the *i*-th eigenvalue of A (with ${\text{λ}}_{1}\leq {\text{λ}}_{2}\leq \cdots \leq {\text{λ}}_{n}$), and $V^{i}\equiv \left(r_{1}^{i}\;,r_{2}^{i}\;,\;\cdots ,r_{n}^{i}\;\right)$ is the eigenvector corresponding to $\lambda_{i}$.

The original TDS matrix A can be presented as a spectrum decomposition $A=U^{Τ}DU=\sum_{i=1}^{n}\lambda_{i}V^{iΤ}V^{i}$, where the major component M of matrix A that corresponds to the largest eigenvalue $\lambda_{n}$is:

(2) $$M\equiv V^{nT}V^{n}=(\begin{array}{cccc} r_{1}^{n}r_{1}^{n} & r_{1}^{n}r_{2}^{n} & \cdots & r_{1}^{n}r_{n}^{n}\\ r_{2}^{n}r_{1}^{n} & r_{2}^{n}r_{2}^{n} & \cdots & r_{2}^{n}r_{n}^{n}\\ \vdots & \vdots & \ddots & \vdots \\ r_{n}^{n}r_{1}^{n} & r_{n}^{n}r_{1}^{n} & \cdots & r_{n}^{n}r_{n}^{n} \end{array}).$$

Because $\lambda_{n}$ is the largest eigenvalue of *A*, the matrix *M* represents the first-order approximation for the links configuration of the original TDS network, where network links strength is quantified by the off-diagonal matrix elements $r_{i}^{n}r_{j}^{n}$ for i≠j, and the relative weight of each node *i* in the network dynamics is represented by the diagonal elements $r_{i}^{n}r_{i}^{n}$.

## 3. Data

**Figure 1.**
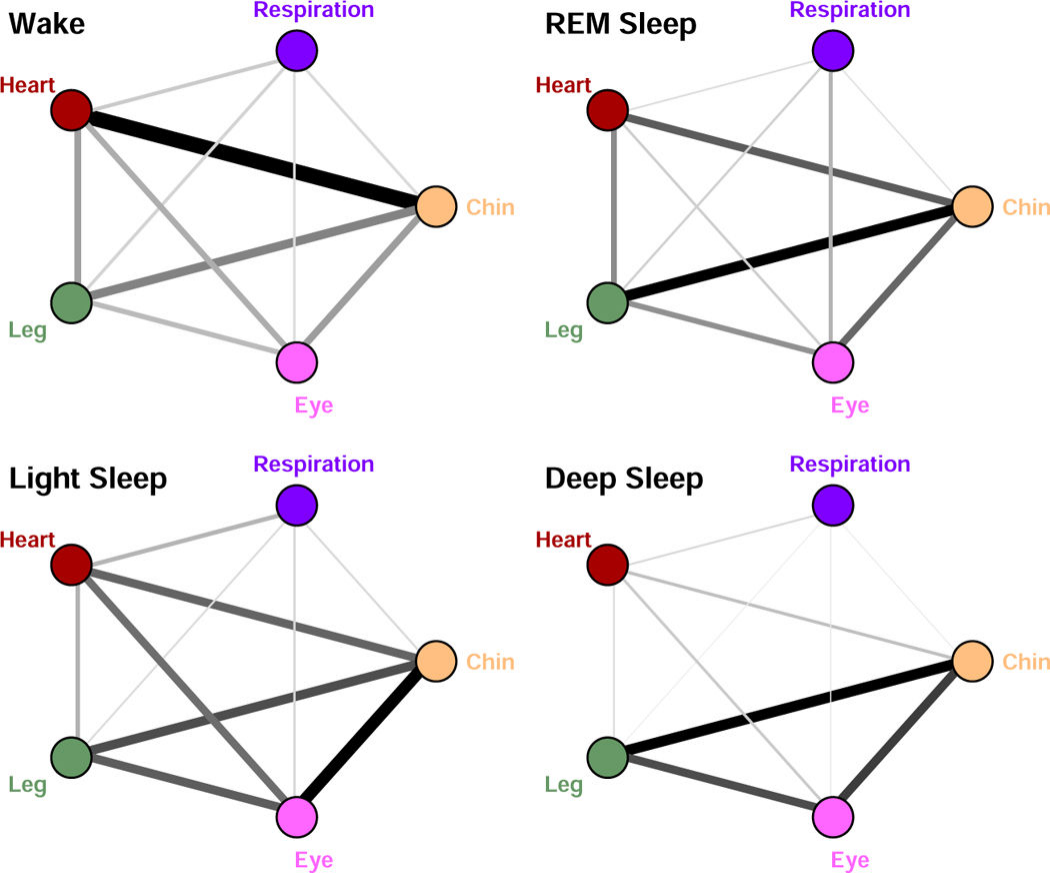
Network of interactions between key organ systems and transitions across different physiologic states. Interactions between organ systems are represented by weighted undirected graphs, where links reflect the strength of dynamic coupling as measured by the stability in the time delay between bursts of activities in the systems output signals (% TDS, see Methods). Darker and thicker links correspond to stronger coupling and higher time delay stability. Results are obtained from 8-hour recordings during sleep by weighted averaging over all segments of different sleep stages pooled from 36 healthy subjects. A pronounced re-organization in the configuration of network links strength is observed with transitions from one sleep stage to another, demonstrating a clear association between network structure and physiologic function.

We analyse continuously recorded multichannel physiological data obtained from 36 healthy young subjects (18 female and 18 male; 20 – 40 years old, average age 29) during night-time sleep [14] (average record duration is 7.8 h). This allows us to track the dynamics and evolution of the network of physiological interactions during different sleep stages and sleep-stage transitions (Fig. 1). We focus on physiological dynamics during sleep as sleep stages are well-defined physiological states, and external influences due to physical activity or sensory inputs are reduced during sleep. Sleep stages are scored in 30s epochs by sleep lab technicians based on standard criteria. We study the electrocardiogram (ECG), respiration, the electrooculogram (EOG), and the electromyogram (EMG) of the chin and leg. In order to compare these very different signals with each other and to study interrelations between them, we extract the following time series from the raw signals: the variance of the EOG and EMG signals in moving windows of 2s with a 1s overlap; heartbeat RR intervals and interbreath intervals are both re-sampled to 1 Hz (1s bins) after which values are inverted to obtain instantaneous heart and respiratory rate. Thus, all time series have the same time resolution of 1s before the TDS method of analysis is applied.

## 4. Results and Summary

We find that different physiologic states are characterized by significantly different average strength of network links as measured by the proportion of data segment with time delay stability (% TDS, see Methods): stronger links during Wake (*≈* 8.4% TDS) and Light Sleep (*≈* 8.4% TDS), and much weaker links in REM (*≈* 6.8% TDS) and Deep Sleep (*≈* 5.5% TDS). This sleep-stage stratification pattern is observed across all individual subjects, indicating a universal behavior related to the underlying mechanism of neural regulation. The observed similarity in the average links strength between Wake and Light Sleep is intriguing, since traditionally physiologic dynamics are considered similar during Light Sleep and Deep Sleep (Non-REM phase), and very different from Wake and REM.

**Figure 2.**
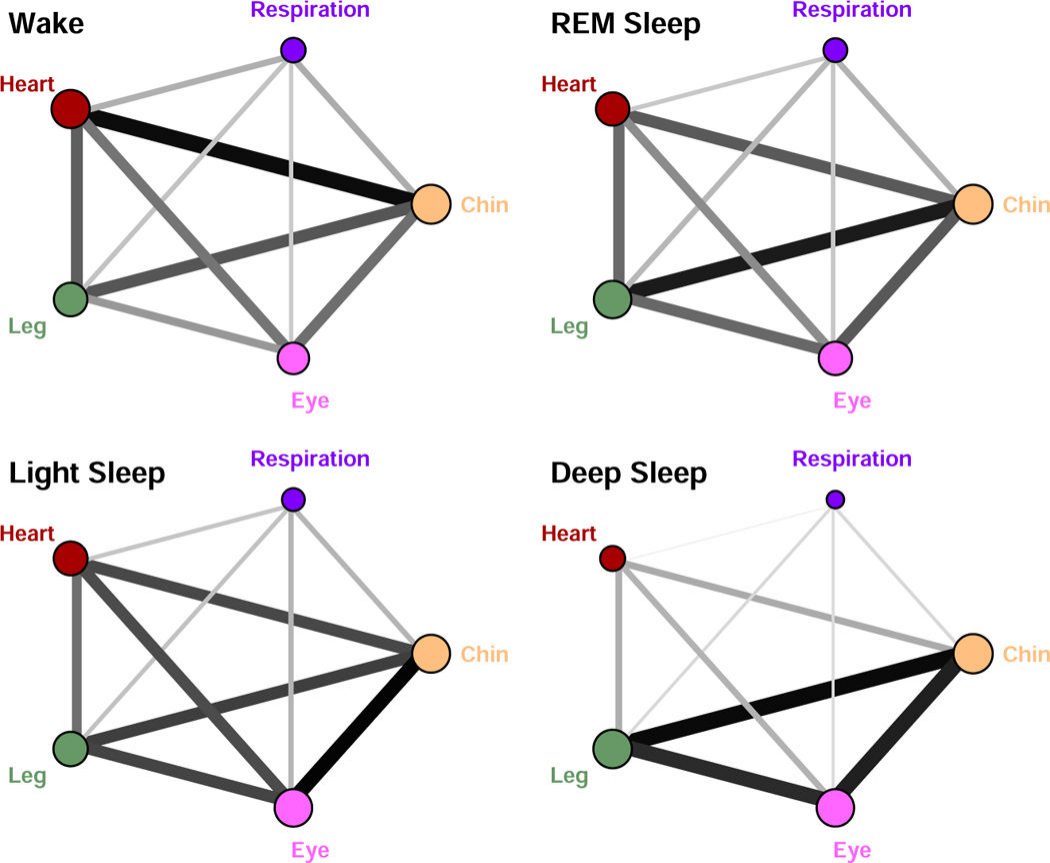
Weighted undirected graphs represent the major component of the networks of organ systems interactions for different physiologic states. The relative contribution of each organ system to the global network dynamics is presented by the size of each network node — the radius of each network node *i* is proportional to the component $r_{i}^{n}$ of the eigenvector *V* ^*n*^ corresponding to the largest eigenvalue λ_*n*_ of the original TDS network for each physiologic state (Fig.1). Links strength (indicated by different line thickness) is proportional to the off-diagonal elements $M_{i,j}=r_{i}^{n}r_{j}^{n}(i\neq j)$ of the major component of the original TDS networks in Fig.1 (see Methods). Graphs show a gradual decrease of the role of heart and respiration in the physiologic network dynamics with transition from wake to REM, to light and deep sleep, while in contrast, the relative contribution of leg and chin muscle tone and eye movement increases.

We find that different physiologic states are characterized by different largest eigenvalues λ_*n*_, which form a pronounced stratification pattern across physiologic states (higher values for Wake and LS, and lower values for REM and DS, Table. 1), indicating transitions in the connectivity strength of the TDS network with physiologic states consistent with earlier observations [1]. Further, our results of the major component analysis of the TDS networks shown in Fig. 1 demonstrate that different physiologic systems have very different relative contribution to global network dynamics, as quantified by the components *r*_*i*_ of the eigenvector *V* ^*n*^ corresponding to λ_*n*_ of the TDS matrix for each state (Table. 1), as well as by the size of network nodes (Fig. 2).

Our analyses show that the relative weight of the heart in network dynamics is higher than respiration. Further, we find that for both heart and respiration, the relative contribution to the global dynamics gradually decreases from Wake to REM, to Light and Deep Sleep, while in contrast, contributions from chin, leg and eye movement increase and are highest during Deep Sleep (Fig. 2, and Table. 1). Notably, the reduced role and link strength of the heart and respiratory system during Light Sleep and Deep Sleep compared to REM and Wake is consistent with earlier findings of reduced sympathetic input and associated loss of long-range auto-correlation in cardiac and respiratory dynamics during Light and Deep Sleep [6, 8, 15, 16].

In summary, we demonstrate that a network approach to physiological interactions is necessary to understand how modulations in the regulatory mechanism of individual organ systems translate into reorganization of physiological interactions across the human organism. We find that the bursting activity in the output dynamics of key organ systems exhibits a robust pattern characterized by a stable time delay that persist in time during a given physiologic state. This pattern is consistently observed in all subjects for the same physiologic state, indicating a universal association between network topology and physiologic function. Specifically, we find that each physiologic state is characterized by a distinct configuration and distribution of links strength, and that transitions from one physiologic state to another are associated with rapid reorganization (within 30s to 1min) in network links strength and configuration. The proposed novel approach allows to dissect dynamic networks and to the quantify relative contributions from different nodes to global network dynamics. This is essential to address a fundamental question in the new field of Network Physiology [1, 2], namely, how physiologic organ systems interact as a network to synchronize and optimize their function to produce health and disease.

## Figures and Tables

**Table 1.** Largest eigenvalues λ_*n*_ and corresponding eigenvectors *V* ^*n*^ of the TDS matrix *A* representing the network of organ interactions shown in Fig. 1 for different physiologic states (Wake, REM, Light Sleep (LS) and Deep Sleep (DS)). Eigenvector components *r*_*i*_(*i* = 1, …, 5) correspond to different organ systems: chin, respiration, heart, leg and eye, respectively.

	$V_{\text{Wake}}^{n}\left(\lambda_{n}=0.37\right)$	$V_{\text{REM}}^{n}\left(\lambda_{n}=0.30\right)$	$V_{\text{LS}}^{n}\left(\lambda_{n}=0.36\right)$	$V_{\text{DS}}^{n}\left(\lambda_{n}=0.27\right)$
*r*_1_: Chin	0.5714	0.5711	0.5421	0.5805
*r*_2_: Respiration	0.2251	0.2124	0.1959	0.1137
*r*_3_: Heart	0.5494	0.4171	0.4307	0.2347
*r*_4_: Leg	0.4251	0.5284	0.4462	0.5601
*r*_5_: Eye	0.3744	0.4189	0.5321	0.5304

## References

[1] Bashan A, Bartsch RP, Kantelhardt JW, Havlin S and Ivanov P Ch 2012 Nat. Commun 3 702 22426223 10.1038/ncomms1705 PMC3518900

[2] Ivanov P Ch and Bartsch RP 2014 Network Physiology: Mapping Interactions Between Networks of Physiologic Networks (Springer International Publishing) chap 10, pp 203–222

[3] Ivanov P Ch, Rosenblum MG, Peng CK, Mietus J, Havlin S, Stanley HE and Goldberger AL 1996 Nature 383 323–327 8848043 10.1038/383323a0

[4] Ivanov P Ch, Amaral LAN, Goldberger AL, Havlin S, Rosenblum MG, Struzik ZR and Stanley HE 1999 Nature 399 461–465 10365957 10.1038/20924

[5] Schmitt DT and Ivanov P Ch 2007 Am. J. Physiol.-Regul. Integr. Comp. Physiol 293 R1923–R1937 17670859 10.1152/ajpregu.00372.2007

[6] Kantelhardt JW, Havlin S and Ivanov P Ch 2003 Europhys Lett 62 147–153

[7] Ivanov P Ch, Bunde A, Amaral LAN, Havlin S, Fritsch-Yelle J, Baevsky RM, Stanley HE and Goldberger AL 1999 Europhys. Lett 48 594–600 11542917 10.1209/epl/i1999-00525-0

[8] Kantelhardt JW, Ashkenazy Y, Ivanov P Ch, Bunde A, Havlin S, Penzel T, Peter JH and Stanley HE 2002 Phys. Rev. E 65 051908 10.1103/PhysRevE.65.051908 12059594

[9] Hu K, Ivanov P Ch, Hilton MF, Chen Z, Ayers RT, Stanley HE and Shea SA 2004 Proc. Natl. Acad. Sci. USA 101 18223–18227 15611476 10.1073/pnas.0408243101 PMC539796

[10] Ivanov P Ch, Hu K, Hilton MF, Shea SA and Stanley HE 2007 Proc. Natl. Acad. Sci. USA 104 20702–20707 18093917 10.1073/pnas.0709957104 PMC2410066

[11] Bartsch RP, Schumann AY, Kantelhardt JW, Penzel T and Ivanov P Ch 2012 Proc. Natl. Acad. Sci. USA 109 10181–10186 URL 10.1073/pnas.1204568109 22691492 PMC3387128

[12] Bartsch RP and Ivanov P Ch 2014 Communications in Computer and Information Science 438 270–287

[13] Bartsch RP, Liu KKL, Ma QDY and Ivanov P Ch 2014 Computing in Cardiology 41 in press PMC4319215 25664348

[14] Kl¨osch G, Kemp B, Penzel T, Schlo¨gl A, Rappelsberger P, Trenker E, Gruber G, Zeitlhofer J, Saletu B, Herrmann WM, Himanen SL, Kunz D, Barbanoj MJ, R¨oschke J, V¨arri A and Dorffner G 2001 IEEE Eng. Med. Biol. Mag 20 51–57 11446210 10.1109/51.932725

[15] Schmitt DT, Stein PK and Ivanov P Ch 2009 IEEE Trans. Biomed. Eng 56 1564–1573 19203874 10.1109/TBME.2009.2014819 PMC2821156

[16] Schumann AY, Bartsch RP, Penzel T, Ivanov P Ch and Kantelhardt JW 2010 Sleep 33 943–955 20614854 10.1093/sleep/33.7.943 PMC2894436

